# Molecular Characterization of Gene Encoding Outer Membrane Protein *loa22* in Pathogenic *Leptospira* Serovars in Iran

**DOI:** 10.1155/jotm/3900663

**Published:** 2024-12-28

**Authors:** Yeganeh Malek Mohammadi, Pejvak Khaki, Mehdi Gharakhani

**Affiliations:** Department of Microbiology, Agricultural Research, Education and Extension Organization (AREEO), Razi Vaccine and Serum Research Institute, Karaj, Iran

**Keywords:** cloning, leptospirosis, *loa22* gene, pathogenic serovars, sequencing

## Abstract

The *loa22* protein is highly conserved among pathogenic *Leptospira* serovars and it is expressed during both acute and chronic infections. The aim of this study was to clone and sequence of the *loa22* protein-encoding gene of *Leptospira* serovars. In this study, 23 pathogenic *Leptospira* serovars and two nonpathogenic *Leptospira* serovars were used. These serovars were obtained from the microbial culture collection of *Leptospira* Reference Laboratory, Department of Microbiology, Razi Vaccine and Serum Research Institute, Karaj, Iran. Three serovars, including *L.* Sejroe Hardjo-bovis, *L.* Grippotyphosa, *L.* Canicola, are used in the preparation of the trivalent vaccine. The *loa22* gene was amplified by specific primers and the PCR products were then purified using kit and were cloned into a pTZ57R/T vector and transformed in competent *E. coli* DH5*α* cells. The cells were then plated onto LB agar containing ampicillin and recombinant colonies subjected to colony PCR to confirm the presence of the *Leptospira*l gene. Positive colonies plasmid vector was isolated from cells by High Pure Plasmid Isolation Kit. The *loa22* gene was detected in all 23 pathogenic serovars, while this gene was not observed in nonpathogenic *L. biflexa.* It was determined that the similarity percentage of the sequenced pathogenic serovars is between 95.5% and 100%. The results concluded that the *loa22* gene was highly conserved among various pathogenic *Leptospira* serovars and can be used to develop an effective recombinant vaccine.

## 1. Introduction

Leptospirosis is a re-emerging infectious disease, caused by pathogenic *Leptospira* species, which can lead to multisystemic involvements and cause high morbidity and mortality in animals and humans [1–3]. About 64 pathogenic and saprophytic species and more than 300 serovars of *Leptospira* have been identified, of which 38 species are pathogenic [4]. The disease is widespread worldwide and is also more prevalent in tropical and humid regions with high rainfall than temperate countries [5]. Studies show that leptospirosis in Iran is more common in the northern provinces [6]. Infection usually occurs through direct contact with the urine of an infected animal or indirectly through contaminated water and soil, and almost any mammal can act as a carrier of *Leptospira* [7].

The spectrum of the disease can vary from subclinical infection, a flu-like febrile disease, or severe systemic potentially fatal disease with hepatic and renal involvement, myocarditis, and extensive vasculitis [8, 9]. During the disease, various organs including the kidneys, liver, meninges, lungs, muscles, and placenta may be damaged [1]. The mortality rate in patients with severe icteric leptospirosis is usually between 5% and 15% [9].

The pathogenesis of *Leptospira* is still not well known, and factors such as adhesins, toxins, and other surface proteins may be involved in pathogenesis [1, 10]. Unlike lipopolysaccharide (LPS), proteins derived from pathogenic *Leptospira* can provide protective immunity versus heterologous *Leptospira* serovars in experimental animal models [11]. The outer membrane (OM) of *Leptospira* has a variety of virulence factors that are involved in the pathogenesis [12, 13], including the OmpA-like protein *loa22*, which is a highly conserved lipoprotein that plays a key role in the virulence of *Leptospira* [14]. The *loa22* protien may play a physiological role in maintaining the integrity of the membrane structure. Like OmpA, it also acts as a multifunctional protein and may be involved in cell adhesion, tissue invasion, and induction of an immune response [11]. In vitro, recombinant *loa22* (*rloa22*) binds to extracellular matrix components such as type I and IV collagen and plasma fibronectin, suggesting that the *loa22* domain may act as an adhesion [15, 16]. The *loa22* is expressed during acute and chronic infections and elicits an immune response in patients, and can also be detected in the serum of human patients. Recently, partial protection has been reported with the *loa22* vaccine against *Leptospira* in some animal models, such as hamsters [17, 18].

Because of the health and economic importance of leptospirosis and also the increasing incidence of this zoonotic disease in different parts of Iran, so the study of *Leptospira* to identify rapid methods of diagnosis and prevention of leptospirosis is essential [16]. Therefore, designing and manufacturing an effective recombinant vaccine to control leptospirosis is very important [6]. On the other hand, due to the importance of early diagnosis of this disease, the existence of a suitable test with high sensitivity and specificity for the correct and early diagnosis of this disease seems necessary. For this purpose, recombinant *Leptospira* proteins can be used as antigens in serological tests as well as its pathogenicity dominant genes in molecular diagnostic tests [19].

Various studies have shown that *loa22* OM protein is present only in pathogenic *Leptospira* and may be used as a candidate for a new vaccine against infection with pathogenic serovars as well as for the development of ELISA for serological diagnosis of leptospirosis [11, 20, 21]. Also, since *loa22* gene is conserved, it is possible to use this gene for accurate and quick molecular diagnosis [22]. The objective of this study was to cloning and sequencing the *loa22* protein-encoding gene in pathogenic serovars of *Leptospira*.

## 2. Materials and Methods

### 2.1. *Leptospira* Serovars and Culture Conditions

In this study, twenty-three pathogenic *Leptospira* serovars and two nonpathogenic *Leptospira* serovars were used (Table 1). These serovars were prepared from the microbial collection of *Leptospira* Reference Laboratory, Department of Microbiology, Razi Vaccine and Serum Research Institute, Karaj, Iran. Twenty-three pathogenic serovars were isolated from cattle and two nonpathogenic serovars from environmental samples. The serovars were inoculated for 5–7 days into the selective culture medium EMJH (Difco, Sparks, USA) supplemented with *Leptospira* enrichment and 10% rabbit serum at 28°C under aerobic conditions [14].

### 2.2. DNA Extraction

The bacterial genome was extracted using phenol-chloroform method and the quality and quantity of the extracted DNA of *Leptospira* serovars were determined by spectrophotometry by nanodrop device (Epoch-BioTek, Winooski, VT, USA) [23].

### 2.3. Primer Design

In this study, specific primers of *loa22* gene (671 bp) were designed to amplification the gene in *Leptospira*l serovars (Table 2).

### 2.4. *loa22* Amplification by PCR

The PCR reaction was conducted in a final volume of 12 μL consisting of 6 μL of master mix (2x Taq^Basic^ PCR Master Mix 2 [BioFACT Co., Korea]), 1 μL of forward primer (10 pmol/μL), 1 μL of reverse primer (10 pmol/μL), 1 μL of genomic DNA containing approximately 100 ng of DNA and 3 μL of sterile deionized water. The thermal cycling protocol for PCR was comprised an initial denaturation at 94°C for 5 min, followed by 35 cycles of denaturation at 94°C for 60 s, annealing at 62°C for 60 s and extension at 72°C for 60 s, with a final extension at 72°C for 10 min. The PCR amplicons were visualized using an ultraviolet (UV) light box after electrophoresis (75 min at 70 V) on a 1% agarose gel.

### 2.5. Cloning of *loa22* Gene

The PCR products were purified by Thermo Fisher Scientific Purification Kit (Thermo Fisher Scientific, the United States). The purified *loa22* genes (from 18 most common serovars), each was independently integrated into pTZ57R/T vector by Thermo Scientific CloneJET PCR Cloning Kit (Thermo Fisher Scientific, US).

After integrating the *loa22* genes into the vector, the recombinant plasmids were transferred to *E. coli* (DH5*α*) susceptible cells by heat shock method. Finally, the bacteria containing the recombinant plasmids were cultured on LB agar medium containing ampicillin and incubated at 37°C for 24 h. After the required time, the growth of recombinant colonies was checked on the culture medium.

The presence of *loa22* gene in recombinant colonies was confirmed by PCR assay. The recombinant plasmids were then purified from the recombinant cells by High Pure Plasmid Isolation Kit (Roche, Germany) and the recombinant plasmids containing the *loa22* gene were sequenced in the Microscience Company, Switzerland.

### 2.6. Nucleotide Sequencing and Homological Analysis

In this study, 18 pathogenic serovars were sequenced. The sequences were compared with the sequences in the GenBank using BLAST program. Finally, the sequences obtained from the present study and similar sequences obtained in the GenBank were compared using the Meg Align program, and also, in order to investigate the phylogenetic relationships of different strains of *Leptospira* based on *loa22* gene, phylogenetic tree and similarity and divergence table of sequences were drawn using this software. All 18 sequenced serovars in our study were registered in NCBI GenBank (Table 1).

## 3. Results

### 3.1. *loa22* Amplification by PCR

**Figure 1:**
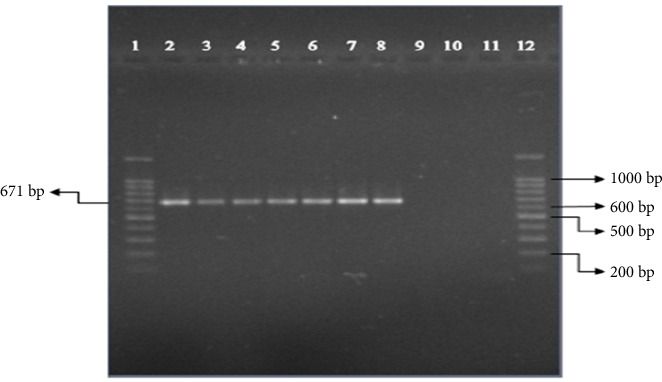
PCR results of seven pathogenic and two nonpathogenic of *Leptospira* studied samples. (1) 100 bp size marker (Bio-Rad); (2) *L. autumnalis* (RTCC 2802); (3) *L. *Canicola (RTCC 2805); (4) *L. *Grippotyphosa (RTCC 2808); (5) *L. icterohaemorrhagiae* (RTCC 2812); (6) *L. pomona* (RTCC 2815); (7) *L. icterohaemorrhagiae* (RTCC 2823); (8) positive control (*L. icterohaemorrhagiae* [RTCC 2837]); (9): *L. biflexa* (RTCC 2819); (10): *L. biflexa* (RTCC 2828); (11) Negative control; (12): 100 bp size marker (Bio-Rad).

As shown in Figure 1, a 671 bp fragment was observed only in pathogenic serovars, whereas saprophytic *Leptospira* lacked this gene.

### 3.2. Cloning of the *loa22* Gene

**Figure 2:**
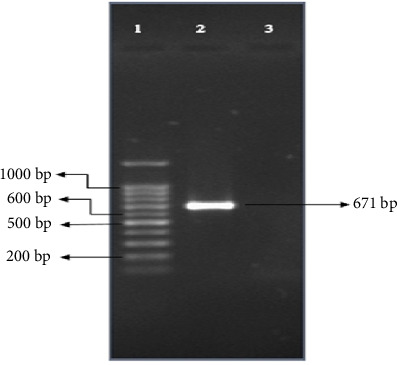
Evaluation of the cloned *loa22* gene in *E. coli* (DH5*α*) by PCR colony. (1) 100 bp size marker (Bio-Rad); (2) *loa22* cloned gene; (3) negative control.

The *loa22* genes were successfully subcloned into pTZ57R/T vector and the recombinant vector, *loa22*-pTZ57R/T, was introduced into competent DH5*α E. coli*.  Figure 2 shows the desired band in the recombinant cell in which the plasmid was transformed with the *loa22* gene.

**Figure 3:**
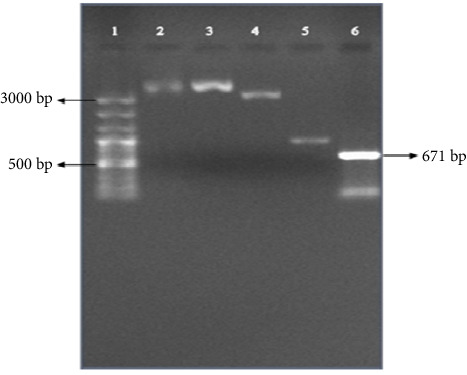
Purified plasmid electrophoresis of *L. interrogans* serovar *Hardjo-bovis* (2810). (1) 100 bp plus size marker (Bio-Fact); (2) plasmid containing *loa22* gene; (3) control 2 or positive control kit; (4) control 1 or negative control kit; (5) PCR fragment control; (6) PCR product (671 bp).

As a result, considering that the length of the pTZ57R/T vector inside the kit was 2886 bp and the desired fragments were 671 bp (Figure 3), so the length of the recombinant plasmid fragments were 3557 bp and also the length of the PCR Fragment control was 953 bp which was inserted into the vector plasmid. So, it created a length of 3839 bp, which was the positive control of the kit. The negative control consisted of a vector plasmid without an inserted fragment with a length of 2886 bp. Consequently, band lengths were observed on agarose gel based on the mentioned sizes (Figure 3).

### 3.3. Sequencing and Homological Analysis of the *loa22* Gene

In the present study, eighteen pathogenic serovars were sequenced and the sequences were analyzed by MegAlign program. Moreover, 3 serovars registered for *loa22* gene in NCBI were compared with all 18 sequenced serovars and genetic similarity surveys were performed on these serovars, which included the *L. australis* (KM435348), *L.* Grippotyphosa (KC311551) and *L. Hardjo*-*prajitno* (MT941858). In order to investigate the phylogenetic relationship of different *Leptospira* serovars based on *loa22* gene, phylogenetic tree and sequence similarity and divergence table were drawn using MegAlign program.

**Figure 4:**
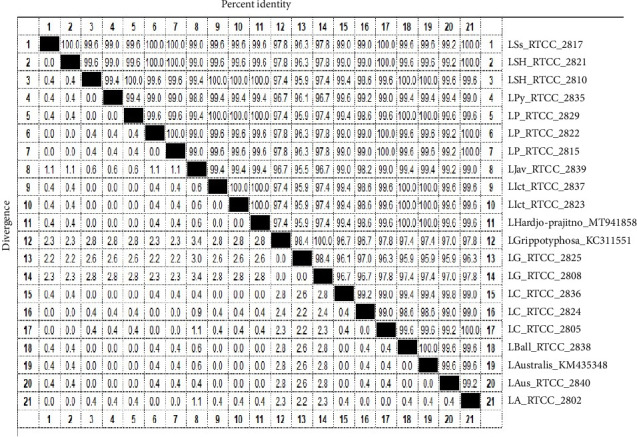
Percentage of similarity and divergence between different sequenced serovars in the present study and *Leptospira* serovars registered in NCBI for *loa22* gene, based on sequence analysis of *loa22* gene using MegAlign software.

According to Figure 4, the minimum similarity between serovars was 95.5 and the maximum was 100%. Also, the sequencing results showed that the similarity between the same serovars was very high (up to 100%).

In general, two *L.* Grippotyphosa serovars (RTCC 2808, 2825) had high similarity (98.4%) with each other, but had the least similarities with the other serovars (Figure 4). Among 18 sequenced serovars, *L. javanica* (RTCC 2839) serovar had the least similarity (95.5%) with *L.* Grippotyphosa (RTCC 2825).

As shown in Figure 4, eighteen sequenced serovars in this study were compared with three serovars registered for the *loa22* gene in the NCBI.

The *L. Hardjo-prajitno* serovar (MT941858) had 100% and 99.6% similarity with our *L. Hardjo-bovis* serovar (RTCC 2810) and *L. Hardjo-prajitno* serovar (RTCC 2821), respectively. Moreover, this serovar had the highest similarity (100%) with different our serovars such as *L. Ballum* (RTCC 2838), *L. pomona* (RTCC 2829), *L. icterohaemorrhagiae* (RTCC 2837, 2823) and had the lowest similarity (95.9%) with native *L.* Grippotyphosa (RTCC 2825).

The *L. *Grippotyphosa serovar (KC311551) had 100% and 98.4% similarity with our *L.* Grippotyphosa (RTCC 2808) and *L.* Grippotyphosa (RTCC 2825), respectively. Furthermore, this serovar was most similar (100%) to our *L. *Grippotyphosa serovar (RTCC 2808). It also showed the lowest similarity (96.7%) with native *L.* Canicola (RTCC 2824, 2836), *L. pyrogenes* (RTCC 2835) and *L. javanica* (RTCC 2839) serovars.

The *L. australis* serovar (KM435348) was 99.6% similar to our *L. australis* serovar (RTCC 2840). In addition, this serovar had the most similarity (100%) with our various serovars including *L. Hardjo-bovis* (RTCC 2810), *L. pomona* (RTCC 2829), *L. icterohaemorrhagiae* (RTCC 2837, 2823) and *L. Ballum* (RTCC 2838) and also had the least similarity (95.9%) with native *L.* Grippotyphosa (RTCC 2825).

The results showed that the similarities did not depend on the serogroups and serovars, and the similarities were even seen up to 100% in different serovars.

**Figure 5:**
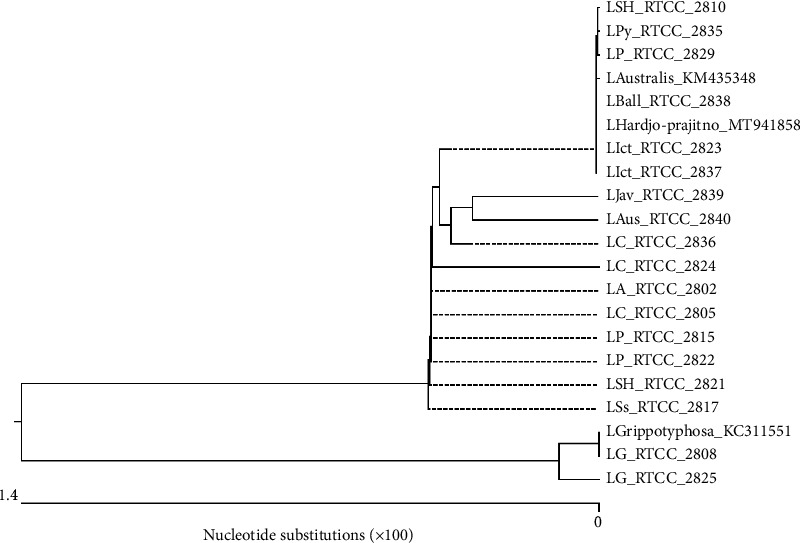
Dendrogram of the similarity of 18 serovars sequenced in the present study with three serovars registered in the NCBI based on the sequence analysis of the *loa22* gene using MegAlign software.

Based on the dendrogram drawn in Figure 5, eighteen sequenced serovars in the present study and three serovars registered for the *loa22* gene in the NCBI were compared and placed in two clusters. Cluster I consisted of 16 sequenced serovars along with two serovars registered in the NCBI including *L. australis* (KM435348) and *L. Hardjo-prajitno* (MT941858). Cluster II included two native *L.* Grippotyphosa serovars (RTCC 2808, 2825) and one NCBI-registered *L.* Grippotyphosa serovar (KC311551). Cluster II was divided into two branches, the first of which was the NCBI-registered serovar *L.* Grippotyphosa (KC311551) and the native *L.* Grippotyphosa serovar (RTCC 2808), which had 100% similarity. The second branch consisted only one native *L.* Grippotyphosa serovar (RTCC 2825), which had 98.4% similarity to other *L.* Grippotyphosa serovars. As a result, native and NCBI-registered *L. *Grippotyphosa serovars were placed in a separate cluster due to their high similarity to each other and had more differences than the other serovars.

## 4. Discussion

Leptospirosis is one of the most important diseases that can be transmitted from livestock to humans and infects more than one million people worldwide each year [1, 24]. The highest disease prevalence is found in temperate and tropical regions, especially in areas with high rainfall, and areas with neutral pH or slightly alkaline soils such as northern Iran [25]. Unfortunately, for various reasons, this disease is not diagnosed correctly. Therefore, rapid and accurate diagnosis of the disease and the distinction of pathogenic species from nonpathogenic is one of the most important measures that must be taken to prevent, control and properly treat the disease [26, 27]. Various approaches including bacteriological and serological methods such as microscopic agglutination test (MAT) and ELISA are used to diagnose leptospirosis, but each has disadvantages. Due to their high sensitivity and accuracy, molecular methods have become suitable candidates for the diagnosis of this bacterium [28]. In addition, despite vaccination against leptospirosis, there are still records of the disease in some parts of the country. *loa22* protein could be a new vaccine candidate for protection against *Leptospira* infection, which requires further studies to confirm the importance of *loa22* in pathogenicity as well as protective activity [11]. Recently, the identification of the OM proteins of pathogenic *Leptospira* has been a very important research topic in *Leptospira*. Among these proteins, *loa22* has been identified in pathogenic *Leptospira*, but not in nonpathogenic *Leptospira*, indicating a possible role for this protein in bacterial virulence [29, 30]. Various studies have shown that *loa22* is expressed during infection and can be detected by patients' serum and also triggers an immune response in patients. It is also a surface protein and provides partial protection in hamsters, and could possibly be considered a suitable candidate for the vaccine [29, 30]. Accurate identification of the dominant serovars in each region is necessary to develop an effective vaccine. Serovars cannot be identified by serology (MAT) and culture methods and also these methods require a lot of facilities. Although the identification of serovars does not seem necessary for treatment, it is important for optimizing vaccine production and epidemiological goals and controlling infection. Most animal vaccines are obtained from inactivated cells or from the cell wall of the pathogenic *Leptospira*. These vaccines produce protection against *Leptospira* by inducing antibodies against LPSs and have disadvantages such as side effects, short-term effects, and incomplete protection against other serovars. The development of molecular methods for the study and identification of common *Leptospira* serovars in an area seems to be important for vaccine development because the immunity provided by the immune system is effective only against contaminating serovars. Therefore, the development of vaccines against the disease should include common serovars in the region to create effective safety, so the detection of serovars is very necessary to optimize the vaccine [31, 32].

In recent years, various serological and molecular researches on *Leptospira* have been performed in Iran, such as determining the genetic pattern of *Leptospira* serovars used in the leptospirosis vaccine made by the Razi Vaccine and Serum Research Institute, Karaj [23, 33, 34]. However, there is still no standard method for rapid and accurate diagnosis of the disease in medical diagnostic laboratories and health centers [35].

The aim of our study was cloning and sequence analysis of the *loa22* protein-encoding gene in *Leptospira* serovars. In the present study, as in other studies, it was found that the *loa22* gene is present only in pathogenic *Leptospira* serovars and not in saprophytic *Leptospira* serovars [21]. In a study conducted by Koizumi and Watanabe in Japan, the presence of *loa22* protein among 17 pathogenic and nonpathogenic *Leptospira* strains was investigated by immunoblot analysis with anti-*loa22* serum [11]. In this study, a strong relationship between pathogenicity and the presence of *loa22* was observed, which indicates the involvement of this protein in the pathogenesis of *Leptospira*. It was also observed that *loa22* is present only in pathogenic serovars [11]. In 2015, Varadarajan et al. in India examined 12 pathogenic *Leptospira* reference serogroups as well as 15 samples by PCR method. *loa22* gene was reported in all 12 pathogenic serogroups, and LipL32 and *ligB* genes were positive in 11 and 7 serogroups, respectively. The *loa22* gene was also identified in 15 samples, but the other two genes were not identified in any of the samples. The results of this study showed that the *loa22* virulence gene could be a diagnostic marker of leptospirosis in dogs and a future vaccine candidate [36]. The results of our study are consistent with the findings of other researchers regarding the presence of *loa22* gene in pathogenic *Leptospira* and its absence in nonpathogenic serovars, indicating that this gene may play an important role in *Leptospira* pathogenesis. However, in Haake's study in the United States, a homologue of the *loa22* gene was observed with 56% sequence homology in *L. biflexa* [37].

In our study, cloning of *loa22* protein coding gene was used in order to obtain a pure gene fragment for better sequencing. In addition, the cloning and expression of this gene can be used for purposes such as designing a positive control in a PCR test, detecting bacteria, or designing a recombinant vaccine. According to a 2015 study in Thailand, the immunogenicity of an antileptospirosis vaccine was evaluated in mice using the OM proteins LipL32 and *loa22*, which are thought to be antigens. The immunogenicity of this vaccine formulation was compared with those induced by LipL32 or *loa22* alone. In this study, using a unique plasmid DNA expressing both LipL32 and *loa22* for vaccination, higher antibody responses was induced than when combining the plasmids containing each gene separately. Also, specific antibody responses against LipL32 (total IgG and IgG_1_) and *loa22* (IgG_1_) were higher in mice that received the two antigens in combination than those vaccinated with one antigen alone. As a result, the immunization induced by these two antigens using chitosan as a DNA transfer system induces a higher immune response and may be useful in developing a better vaccine for leptospirosis [17]. In 2010, Zhang et al. conducted a study in China in which a vector was made and *loa22* was artificially expressed in *E. coli* BL21 (DE) pLysS cells. Their study exhibited that *loa22* protein mediated a direct cytotoxic effect on NRK52E cells in a dose-dependent manner [38]. In 2014, Ye et al. tested four recombinant *Leptospira interrogans* proteins, rLipL21, *rloa22*, rLipL32, and rLigACon4-8, to evaluate their potential for use as antigens in the diagnosis of equine leptospirosis. In their study, it was found that the use of four antigens in ELISA has sensitivity and specificity and this test is easy to perform and also the results are consistent with the standard results of the *Leptospira* MAT test [39]. In our study, 18 pathogenic serovars of *Leptospira* were used for cloning and these serovar was successfully cloned using the TA cloning kit.

The analysis of the sequencing results in the present study showed a very high similarity of the *loa22* gene among serovars, and these similarities, both in the native serovars and in the serovars registered in NCBI, were not dependent on the serovars and serogroups, which indicates the stability of this gene. In 2014, Kaur et al. in India cloned and sequenced the three genes *lipL41* (1088 bp), *loa22* (608 bp) and LipL21 (581 bp) *L. interrogans*. The results disclosed that sequences of the *loa22* gene *L. interrogans* serovar Grippotyphosa and LipL21 gene of *L. interrogans* serovar Canicola were conserved in nature, but the sequence of the *lipL41* gene of *L. interrogans* serovar Grippotyphosa exhibited changes and differences in the nucleotide sequence that contribute to the evolution of serovars [20]. The results of our study are consistent with those of Kaur et al. [20]. In the present study, the results of *loa22* gene sequencing in 18 pathogenic *Leptospira* serovars showed that the minimum similarity between different serovars is 95.5% and the maximum is 100%.

The results of sequencing the native serovars showed that the similarities and differences between the serovars are not dependent on the serogroup and serovar, so that based on the *loa22* gene, the different native serovars had very high similarities even up to 100% with each other, for example: each of the serovars *L.* Sejroe Hardjo-bovis, *L. pomona*, *L. icterohaemorrhagiae*, *L. *Canicola and *L. *Sejroe, *L. Ballum* and *L. autumnalis* had 100% similarity with some other serovars.

Our study showed that *loa22* is a stable and conserved gene among different *Leptospira* serovars and has a high degree of conservation. Moreover, according to the results of the present study, this gene can be used in the cloning and expression of a recombinant antigen, so it can be used in the preparation of an effective and efficient recombinant vaccine, as well as in serological diagnostic kits such as ELISA.

## 5. Conclusion

The *loa22* gene was present in pathogenic *Leptospira* serovars but not in nonpathogenic serovars. Based on the results of this study, it can be concluded that the similarities based on *loa22* gene are not dependent on serogroups and serovars and there are very high similarities between the different serovars studied. Sequencing results showed a high percentage of similarity of *loa22* gene in pathogenic *Leptospira* serovars (minimum similarity 95.5% and maximum similarity 100%), which indicates that *loa22* is a highly conservative gene. Due to these characteristics, this gene can be considered a suitable candidate for vaccine against pathogenic *Leptospira* serovars.

## Figures and Tables

**Table 1:** *Leptospira* serovars used in this study.

Number	Serogroups	Serovars	RTCC	Accession numbers
1	*Autumnalis*	*Autumnalis* ∗	2802	OP038304
2	Canicola	Canicola∗	2805	OL689841
3	Grippotyphosa	Grippotyphosa∗	2808	OM913538
4	*Sejroe*	*Hardjo-bovis* ∗	2810	OP038310
5	*Icterohaemorrhagiae*	*Icterohaemorrhagiae*	2812	No data
6	*Pomona*	*Pomona* ∗	2815	OP038305
7	*Sejroe*	*Serjae* ∗	2817	OP038306
8	*Semanerga*	*Patoc*	2819	No data
9	*Sejroe*	*Hardjo prajitno* ∗	2821	OM913537
10	*Pomona*	*Pomona* ∗	2822	OP038311
11	*Icterohaemorrhagiae*	*Icterohaemorrhagiae* ∗	2823	OM913536
12	Canicola	Canicola∗	2824	OP038312
13	Grippotyphosa	Grippotyphosa∗	2825	OP038313
14	*Semanerga*	*Patoc*	2828	No data
15	*Pomona*	*Pomona* ∗	2829	OP038314
16	*Autumnalis*	*Autumnalis*	2830	No data
17	*Malaysia*	*Malaysia*	2831	No data
18	*Pyrogenes*	*Pyrogenes* ∗	2835	OP038307
19	Canicola	Canicola∗	2836	OP038315
20	*Icterohaemorrhagiae*	*Icterohaemorrhagiae* ∗	2837	OP038316
21	*Ballum*	*Ballum* ∗	2838	OP038308
22	*Javanica*	*Javanica* ∗	2839	OP038317
23	*Australis*	*Australis* ∗	2840	OP038309
24	*Laitype lanylokowii*	*Laitype lanylokowii*	2841	No data
25	*Sejroe*	*Hardjo-bovis*	2843	No data

^∗^The *loa22* genes from these serovars were cloned and sequenced.

**Table 2:** Sequence and specificity of *loa22* gene specific primer in this study.

Primer sequence (5′-3′)	Primer length	Fragment length (bp)	Tm	GC%	Reference
F: CGGCCTTTTGAAAGATCGAATTG	23	671	58.87	43.48	[21]
R: ACACTCTGATACCAAACCCCT	21		57.87	47.62	

## Data Availability

The data that support the findings of this study are available from the corresponding author upon reasonable request.
